# Solar Wind Turbulence and Complexity Probed with Rank-Ordered Multifractal Analysis (ROMA) †

**DOI:** 10.3390/e26110929

**Published:** 2024-10-30

**Authors:** Marius Echim, Costel Munteanu, Gabriel Voitcu, Eliza Teodorescu

**Affiliations:** 1 Royal Belgian Institute for Space Aeronomy, Avenue Circulaire 3, 1180 Bruxelles, Belgium; 2 Institute of Space Science INFLPR Subsidiary, Atomiștilor 409, 077125 Măgurele, Romania; gabriel.voitcu@spacescience.ro (G.V.); eliza.teodorescu@spacescience.ro (E.T.)

**Keywords:** solar wind, turbulence, complexity, intermittency, multifractal analysis, rank-ordered multifractal analysis

## Abstract

The Rank-Ordered Multifractal Analysis (ROMA) is a tool designed to characterize scale (in)variance and multifractality based on rank ordering the fluctuations in “groups” characterized by the same mono-fractal behavior (Hurst exponent). A range-limited structure-function analysis provides the mono-fractal index for each rank-ordered range of fluctuations. We discuss here two examples of multi-scale solar wind turbulence and complexity where ROMA is applied on the following: (a) data collected by Ulysses spacecraft in the fast solar wind, outside the ecliptic, between 25 and 31 January 2007, at roughly 2.5 Astronomical Units (AU) from the Sun, in the Southern heliosphere, at latitudes between −76.5 and −77.3 degrees, and (b) slow solar wind data collected in the ecliptic plane by Venus Express spacecraft, at 0.72 AU, on 28 January 2007. The ROMA spectrum of fast solar wind derived from ULYSSES data shows a scale-dependent structure of fluctuations: (1) at the smallest/kinetic range of scales (800 to 3200 km), persistent fluctuations are dominant, and (2) at the inertial range of scales (10^4^ to 2 × 10^5^ km), anti-persistent fluctuations are dominant, but less clearly developed and possibly indicative for the development of instabilities with cross-over behavior. The ROMA spectrum of the slow solar wind derived from Venus Express data, suggests a different structure of turbulence: (1) fully developed multifractal turbulence across scales between 5 × 10^4^ and 4 × 10^5^ km, with the Hurst index changing from anti-persistent to persistent values for the larger amplitude magnetic fluctuations; (2) at the smallest scales (400 to 6400 km), fluctuations are mainly anti-persistent, and the ROMA spectrum indicates a tendency towards mono-fractal behavior.

## 1. Introduction

Dynamical complexity is generally considered the physical phenomenon emerging from the mutual nonlinear interaction between (large number of) components of a multi-degrees of freedom dynamical system exhibiting a variety of dynamical manifestations over a large range of spatial-temporal scales [1]. The global dynamical behavior of the system is significantly different than the sum of the component dynamics. The solar wind, the supersonic stream of plasma filling the entire heliosphere and emerging permanently from the Sun’s corona, is known as a turbulent system exhibiting complex behavior and interactions over a large spectrum of scales. Charged particles with broad energy spectra, waves, coherent dynamical structures, pseudo-equilibrium plasma states, etc., populate the solar wind and contribute, individually and mutually coherently, to its complex dynamical features. A fleet of spacecraft probes or probed the solar wind in the ecliptic plane (e.g., Helios, STEREO, ACE, Parker Solar Probe) or outside it (e.g., Ulysses, Solar Orbiter) and collect in situ data on the solar wind’s structure and variability. Voyager crossed the heliopause—the outer boundary of the heliosphere and implicitly of our solar system—and exited into the interstellar medium [2]. Numerous studies identified turbulent properties for various states of the solar wind, fast and slow, at various radial distances and latitudes (for a general review on solar wind turbulence, see, e.g., [3].

Solar wind turbulence bears similarities but also significant differences compared to neutral fluid turbulence. The concept of an energy cascade, which extends over scales spanning several decades, ensuring the transfer of energy from larger (injection) scales to smaller (dissipation) ones, is a classical (fluid turbulence) paradigm adopted for space plasmas [3]. One notable specific feature of space plasmas is, however, the presence of the magnetic field, which is itself involved in the turbulent transfer of magnetohydrodynamic energy but also introduces additional effects, like the anisotropy, rendering the task to quantify space plasma turbulence properties quite difficult. The magnetic field also introduces additional venues for turbulent energy transport and dissipation.

Two fundamental assumptions of the classical Kolmogorovian model of turbulence, i.e., (i) a constant energy transfer rate over all scales and (ii) space-filling interacting non-linear structures, are generally not satisfied in the solar wind (see, e.g., [4]). Indeed, the transfer of energy between scales is characterized by fragmentation and intermittency. Self-similarity is also broken, and higher-order data analysis tools are thus required to capture the complexity of space plasma variability. The Probability Density Functions (PDFs) and their moments capture departure from Gaussianity and self-similarity and help quantify intermittency. The multifractal analysis of data collected in the solar wind provides a hint on the topology of the energy transfer and opens the way to probe various models of non-space filling turbulence (e.g., [5,6]).

In this study, we investigate the turbulent properties of the solar wind using a data analysis method that searches for multifractal behavior based on a rank ordering technique [7]. This technique was specifically invented to overcome the limitations of the standard multifractal analysis and was applied in various contexts, from neutral fluids to astrophysical systems; for a review, see [8].

## 2. Multifractal and Rank-Ordered Multifractal Approach (ROMA)

Fractals and multifractals are introduced as tools to investigate natural phenomena and capture their multi-scale (sometimes self-similar) structure like river networks, systems of Earth’s faults and fractures, various physical processes like lightning and frost formation, mineral crystallinity in rocks, and dendrite formation in chemical reactions, etc., [4,9,10]; see Sornette [11] for a discussion. They are also used to investigate the structure of solar wind turbulence and intermittency from in situ data analysis [12,13]. Multifractals allow for an intuitive understanding of turbulent multiplicative and cascading processes leading to the transfer of energy in a hierarchic structure aggregating larger to smaller scales. The paradigm of a cascade of energy, first coined by Richardson [14], includes two key elements: (1) a hierarchy of mother (level *n*)–daughter (level *n +* 1) structures (e.g., eddies in neutral fluid turbulence), fragmenting into smaller and smaller pieces, from larger to smaller scales, and (2) the rate of energy transfer from the mother to the daughter. In fractal analysis, the latter is linked to the concept of a density measure [15]. Data analysis tools based on multifractals help decipher, through the “optics” of such a multiplicative process, the structure of the energy cascade in the turbulent solar wind. Such tools are particularly helpful in characterizing the degree of fragmentation/irregularity generally linked to intermittency; see, e.g., [16,17].

A key feature of the multifractal analysis is the generalization of the geometrical concept of dimension, which helps to describe the density measure (or energy transfer rate) [18] and the multi-scale structure of the hierarchical mother—daughter cascading over many consecutive generations (e.g., [19]). The fractal structure of neutral fluid turbulence can be approached from an analysis of the Navier–Stokes equation (ref. [19], see a recent review by Dubrulle [17]). Several methods are proposed to obtain the multifractal spectrum, which gives the strength of singularities, f(α) [20].

However, multiplicative processes can also be characterized by the moments of order *q* of the defined measure and their scale behavior, $\langle \epsilon^{q}\rangle$ (the brackets indicate ensemble averaging). The exponents revealed by the scaling of the moments, $\langle \epsilon^{q}\rangle \sim l^{\zeta (q)}$ define a series of generalized dimensions, Dq=ζ(q)(q−1), where D_0_ is the fractal dimension of the support measure, D_1_ is the information dimension, and D_2_ is the correlation dimension; it was however demonstrated that for multifractals there is an infinite number of generalized dimensions for *q* > 0 [21,22]. The moment order *q* explores negative and positive values, similarly to a microscope inspecting different regions of singularities [21,23].

A key technical step in performing a multifractal analysis of data collected in the solar wind is the definition of the measure on which the singularities are searched. Different measures emphasize various effects and physical processes [9,15,24]. A popular approach is the Partition Function (PF) multifractal method, which defines a measure based on the first moment of magnetic field increments [16]:

(1) $$\varepsilon \left(x_{i},l\right)\equiv \left|B\left(x_{i}+l\right)-B\left(x_{i}\right)\right|$$

where B denotes one of the magnetic field components (B_R_, B_T_, B_N_ in case of Ulysses data—in the radial, tangent, and normal directions) or the intensity |B|, separated from a position *x_i_* by a distance *l*. The analysis is applied to the entire time series, which is decomposed into segments of size *l*. Each segment is associated with a turbulent eddy. Furthermore, for each *i*th eddy of size *l,* one associates a probability measure defined by the following:

(2) $$p\left(x_{i},l\right)\equiv \frac{\varepsilon \left(x_{i},l\right)}{\sum_{i=1}^{N}\varepsilon \left(x_{i},l\right)}=p_{i}\left(l\right)$$

In this paradigm, *p_i_(l)* can be interpreted as the local probability (in *x_i_*) that the fraction of energy ε is transferred to an eddy of size *l*. In solar wind studies, it is generally assumed that the Taylor hypothesis is satisfied such that, at a given position *x*, the temporal scales, Δ*t*, can be interpreted as the spatial scales, $l=v_{SW}∆t$, with $v_{SW}$ the average solar wind speed.

The analysis of intermittency with multifractals based on the structure and/or partition function approaches reveals the statistics of the full set of fluctuations, like in (1) and (2) above (see, e.g., [19]). However, the scaling properties derived from such approaches are largely determined by the smaller amplitude fluctuations, which are the most numerous. On the other hand, the rarer but larger fluctuations, although less dominant, are relevant for the scaling properties over the full range of scales. Additionally, universality properties can be probed by a full collapse of probability density functions at all scales on a single master curve. Based on this type of argument, Chang and Wu [7] propose a new technique for the multifractal analysis, closely linked to the concept of scale invariance and rank ordering of fluctuations, called the Rank-Ordered Multifractal Analysis (ROMA).

A key feature of ROMA is that it explores the singular nature of the subdominant fluctuations *at all analyzed scales*. Recall that the multifractal analysis based on the partition function extracts the multifractal spectrum for *the convergence range of scales,* i.e., those scales for which the partition function as a function of scale for the defined measure is linear in the log-log representation for all moment orders *q*. Thus, the first step in ROMA is to isolate ranks of fluctuations with similar fractal (statistical) properties, “collected” from all analyzed scales. This is achieved by grouping the fluctuations according to the range of their scale size. If following [7], we consider the fluctuating magnetic field B(t), then we can form similarly with (1), a series of scale-dependent differences:

(3) $$\delta B\left(t_{i},\tau \right)\equiv \left|B\left(t_{i}+\tau \right)-B\left(t_{i}\right)\right|$$

For a range of time scales τ, corresponding to spatial scales *l = V_SW_* × *τ*, when the Taylor hypothesis is satisfied, with *V_SW_* being the solar wind speed. Then, the probability distribution functions are obtained as normalized histograms of fluctuations at all scales, *P(δB,τ)*.

We note that the PDFs of the fluctuating field exhibit self-similarity (mono-fractal) properties if *P(δB,τ)* for the entire range of scales τ collapse onto one single scaling function P_s_ according to the following scaling formula:

(4) $$P\left(\delta B,\tau \right)\tau^{s}=P_{S}\left(\frac{\delta B}{\tau^{s}}\right)$$

If this is the case, the dynamical behavior of the process leading to the observed fluctuation is considered mono-fractal, characterized by the fractal (Hurst) index s. The scaling relation (4) is also known as the one-parameter (re)scaling and has been tested for space plasmas by [25,26]. Full rescaling (over all scales) of PDFs is equivalent to successfully performing a Dynamic Renormalization Group coarse-graining step in the vicinity of a critical point [1,27].

In practice, the collapse of the PDFs may be “partial,” i.e., only parts of the PDFs collapse on the master curve *P_s_*. In this case, it is considered that the dynamical process is multifractal and the scaling factor *s* in (4) depends on the scaled sizes of fluctuations, $s=s\left(Y\right)$ with $Y=\frac{\delta B}{\tau^{s}}$. The multifractality is then estimated from standard structure-function analysis.

The structure functions of different orders *q* for a given scale τ, $S_{q}\left(\tau \right)$, are calculated from the moments of the probability distribution functions:

(5) $$S_{q}\left(\tau \right)=\langle {\left|\delta B\left(\tau \right)\right|}^{q}\rangle =\int_{0}^{{\delta B}_{max}}{\left|\delta B\left(\tau \right)\right|}^{q}P\left(\delta B,\tau \right)d\delta B$$

where <…> indicates ensemble averaging and ${\delta B}_{max}$ is the maximum value of fluctuations computed with (3) for the scale τ. The structure functions exhibit in general power law scaling, $S_{q}\left(\tau \right)\sim \tau^{\zeta_{q}}$. The existence of such power law behavior is viewed by [28] as a possible manifestation of the dynamical complexity and phase space dynamics in the vicinity of a critical point (see also [27]), where the linear transformation of the quantities *S_q_* and τ of the form $S_{q}\to e^{a_{S}}S_{q}$ and $\tau \to e^{a_{\tau }}\tau$ leads to the invariant $\frac{s}{\tau^{\left(\frac{a_{S}}{a_{\tau }}\right)}}$ . It results in one could formally write $\zeta_{q}=\frac{a_{S}}{a_{\tau }}$.

When the scaling exponent is a linear function of q:

(6) $$\zeta_{q}=sq$$

The process is self-similar/mono-fractal with fractal dimension (or Hurst exponent) *s*. In practice, the scaling exponent $\zeta_{q}$ is evaluated from the slope of the log-log representation of $S_{q}\left(\tau \right)$ for all scales *τ* and all orders *q*. We notice also that in the definition of the structure-function (5), the statistics and scaling are dominated by the most numerous fluctuations, as warned at the beginning of this section. A disadvantage discussed by [7] is that the structure functions defined by (5) are usually divergent for negative orders *q*. When the mono-fractal scaling is not observed and (6) is invalidated, the nonlinearity of $\zeta_{q}$ is generally recognized as a hallmark of intermittency. Several physical mechanisms are considered, including the classical refined similarity hypothesis by Obukov and Kolmogorov [29,30]. For a recent review on solar wind intermittency, see [31].

ROMA is a complementary approach that avoids divergence for negative moment orders and also allows for analysis over the entire range of scales. The first step in ROMA is to group the fluctuations computed with (3) as a function of their scaled size $Y=\frac{\delta B}{\tau^{s}}$, at all analyzed scales τ. However, *s* is not known a priori. Indeed, it is assumed that for each subinterval ΔY there is an index *s*(ΔY) such that all fluctuations in the respective subinterval obey the mono-fractal scaling (6). The question is, of course, how to find the values *s*(ΔY) for the entire range of scaled fluctuations Y.

The answer to this question is central for ROMA, and Chang and Wu [7] describe a procedure to achieve this task. One starts by computing the rank-ordered structure function (ROSF) for the first range of scaled values ΔY_1_, defined between Y_1_ and Y_2_:

(7) $$S_{q}\left(\tau \right)=\langle {\left|\delta B\left(\tau \right)\right|}^{q}\rangle =\int_{a_{1}}^{a_{2}}{\left|\delta B\left(\tau \right)\right|}^{q}P\left(\delta B,\tau \right)d\left(\delta B\right)$$

where $a_{1}=Y_{1}\tau^{s}$ and $a_{2}=Y_{2}\tau^{s}$. The ROMA solution for the interval [Y_1_,Y_2_] is that value of *s* for which the mono-fractal/self-similar scaling is strictly satisfied:

$$S_{q}\left(\tau \right)\sim \tau^{sq}$$

The procedure then continues for the other bins ΔY defined in the space of scaled size fluctuations, until the entire rank-ordered multifractal spectrum is computed. If such a full ROMA spectrum is found, it identifies the rescaling factors for which the PDFs collapse onto one master curve identifying a universal process, possibly a fixed point in a path to criticality [1].

Below we discuss briefly two implementations of the basic principles described above allowing the practical computation of such ROMA solutions. Note that these implementations share the same technical principles and are built in two different programming environments: the approach ODYN is available from a Python code-based Jupyter Notebook library [32]; the approach INA is available from a GUI-based MATLAB standalone Interactive Nonlinear Analysis (INA) library [33].

**Figure 1:**
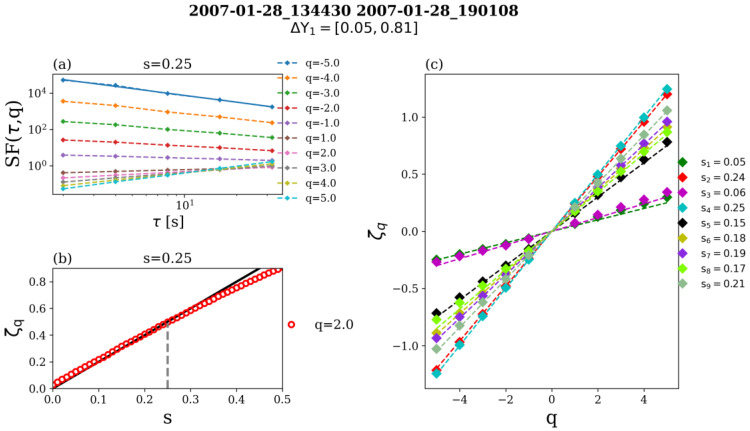
Illustration of the ROMA procedure applied on Venus Express data in the solar wind. (**a**) Range limited structure functions (7) computed for the first bin of scaled fluctuations ΔY_1_ = [0.05, 0.80], ten orders q, from q = −5 to q = +5 and a value assumed a priori s = 0.25; scales from τ_1_ = 2 s to τ_4_ = 64 s are considered. (**b**) Structure-function scaling indices ζ(s,q) computed for $s\in \left[0,0.5\right]$ and q = 2. The intersection between the computed ζ_q_ (in red) and the ζ_q_ = sq line (in black) is marked by the vertical dashed gray line at s = 0.25. (**c**) the slopes ζ(s,q) as a function of q for all values s and for each q for ΔY_1_. The ROMA solution for ΔY_1_ is determined from the best linear fit of ζ_q_(q) lines. In this case, the solution is equal to s = 0.25.

*Computing ROMA with an approach available from the ODYN library.* This approach is documented in [32] and available from the Python library called “Open source library for turbulence and nonlinear dynamics (ODYN)”. For each order *q* and each bin ΔY the procedure runs iteratively for all values of *s* between 0 and 1, with a chosen step, e.g., 0.1, and computes the range-limited structure functions defined by (7) for all analyzed scales. The scaling behavior is extracted from a linear fit in the log-log representation of $S_{q}\left(s,\tau \right)$ versus τ, as shown in Figure 1a, following:

$$S_{q}\left(s,\tau \right)\sim \tau^{\zeta \left(q,s\right)}$$

For each order *q* and each value considered for *s*. In practice, we follow [7] and represent graphically the curve *ζ(s,q)*, derived as described above, and search for its geometrical intersection with the straight line *ζ = qs*, as shown in Figure 1b, for q = 2 and a selected range of s values from 0 to 0.5. If they exist, such intersections result in values of s corresponding to each moment order *q* [34].

Further, for all values of s determined in the previous step, ζ_q_ is retrieved (as shown in Figure 1a for s = 0.25) and the linearity of ζ_q_ vs. q (indicated by colored markers in Figure 1c) is then determined on the basis of a χ^2^ test of linear fits. The value of s which best linearizes the ζ_q_(q) function (i.e., minimum χ^2^) is chosen as the ROMA scaling exponent, *s*, for the analyzed ΔY bin. In this approach, the computation based on finding the intersection shown in Figure 1b is performed iteratively for each value of *q*.

Should there be more than one solution for a given Δ*Y* and a given *q*, we keep the solution that linearizes better the relationship *log(S_q_(τ))* vs. *log(τ)*. If the solution for the given range of scaled size fluctuations, Δ*Y*, and various orders *q* are not the same, we keep the one that satisfies better the mono-fractal relationship: *ζ(s,q) = qs*. The ROMA spectra were calculated with this (classical) approach applied to VEX data in the solar wind.

**Figure 2:**
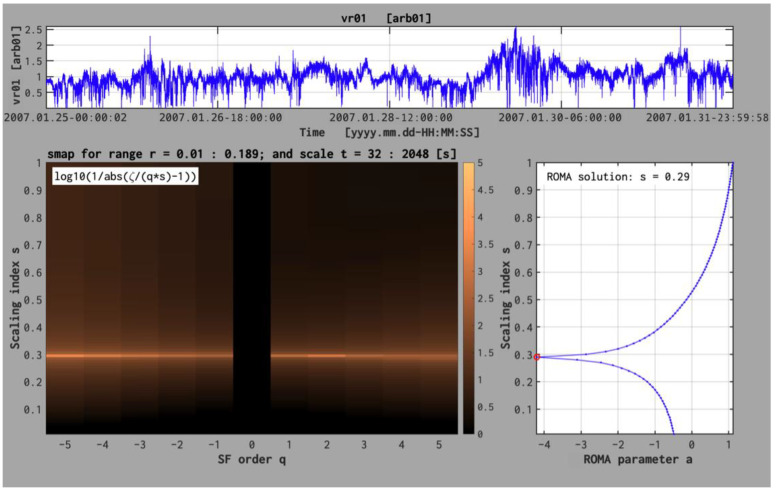
The ROMA approach implemented in INA library [33,35] exemplified for a bin of scaled fluctuations ΔY = [0.01,0.189] and all the moment orders q (from −5 to +5). The upper panel shows the magnetic field energy, ${\left|B\right|}^{2}$, measured by Ulysses between 25 and 31 January 2007. The lower left panel shows the color-coded two-dimensional map of the function log_10_g(q,s); the “brighter” color indicates the maximum of this function, which identifies the ROMA solution for that corresponding q. The right panel shows the result of the global minimization procedure applied for the fluctuations in the bin ΔY = [0.01,0.189]. The procedure minimizes the function $f\left(q,s\right)={\left|\zeta \left(q,s\right)-qs\right|}^{2}$ for 100 values of s between 0 and 1 and all the moments q between −5 and +5.

*Computing ROMA with an approach available from the INA library.* This approach is documented in [33] and available from the MATLAB library called “Interactive Nonlinear Analysis (INA) toolbox.” Munteanu [35] realized that there is a quantitative way to detect the geometrical intersection mentioned above. Indeed, the function:

$$g\left(q,s\right)={\left(abs\left(\frac{\zeta \left(q,s\right)}{qs}-1\right)\right)}^{-1}$$

takes a maximum value when *ζ*(*q,s*) is closest to a linear function in *q*, *ζ*(*q,s*) =*sq*. Thus, one can apply a parametric survey of *g*(*q,s*) to find the value of *s,* which maximizes this function; this maximal value is the ROMA solution for the considered moment order *q*, the considered range of scales, and the considered scaled size fluctuations bin ΔY. An example is shown in Figure 2.

Starting from the quantitative selection of a ROMA solution based on the function *g(q,s)* in [35], an alternative, relatively more compact strategy was tested and implemented to compute the scaling exponents *s* and find the ROMA spectrum based on a minimization procedure. Indeed, in this approach, for each bin of the scaled size fluctuations, ΔY, one iterates over all values of *s* between 0 and 1 (in general one assumes a step equal to 0.1) to compute the range-limited structure function (7) and determine the slope *ζ*(*s,q*) for each *s* and all moment order *q*. Then, one searches for which value of *s* the function $f\left(q,s\right)={\left|\zeta \left(q,s\right)-qs\right|}^{2}$ takes the minimum value for all considered order moments, *q*.

Figure 2 illustrates the two-dimensional map of log10g*(q,s)*, calculated for one hundred values of *s* between 0 and 1, and the first bin of scaled fluctuations, ΔY=[0.01, 0.189]. The map is derived with (3) applied on Ulysses magnetic field data recorded between January 25 and January 31, 2007. This bin collects fluctuations over a scale range between τ = 32 s and τ = 2048 s. The lower-right panel of Figure 2 shows a sum over *q* of all values of the function *f,* labeled parameter *a*:

$$a\left(s\right)=\sum_{q=-5}^{q=+5}f\left(q,s\right)$$

The value of *s* for which parameter *a* takes a minimum value is considered the solution of the ROMA spectrum in that respective bin. An advantage of this approach is that the procedure is applied a single time, for one bin, ΔY. The ROMA spectra computed for Ulysses data and discussed further are derived with the INA implementation [33,35].

**Figure 3:**
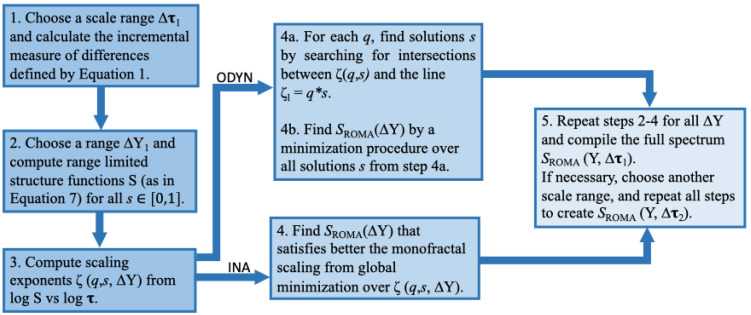
Schematic diagram showing the main steps to calculate the ROMA spectrum. The two implementations—INA and ODYN—are illustrated.

The technical steps to be followed in both strategies adopted to compute a rank-ordered multifractal analysis (ROMA) spectrum are summarized schematically in the diagram shown in Figure 3.

## 3. Solar Wind Rank-Ordered Multifractal Spectra Calculated Outside and in the Ecliptic Plane at 2.5 AU and 0.72 AU

In this section, we discuss a rank-ordered multifractal analysis applied on data from Ulysses, outside the ecliptic at 2.5 AU in *fast solar wind* conditions, and on data from Venus Express (VEX) in the ecliptic plane, close to Venus, at 0.72 AU in *slow solar wind* conditions. The ODYN-based approach is applied to Venus Express data, and the INA-based approach is applied to data from Ulysses.

### 3.1. ROMA and Solar Wind Turbulence and Complexity at 2.5 AU, Outside the Ecliptic

We selected magnetic field data collected at the solar minimum, in the *fast solar wind* (SW speed is of the order of 700 km/s) by Ulysses between 25 and 30 January 2007. Our analysis of Ulysses data focus on three different ranges of scales: small, intermediate, and large, which are defined, as explained below, based on the multi-scale behavior of the flatness. The flatness is defined as a normalized kurtosis [4,36]:

$$K\left(\tau \right)=\frac{\langle {\left|\delta B\left(\tau \right)\right|}^{4}\rangle }{{\left(\langle {\left|\delta B\left(\tau \right)\right|}^{2}\rangle \right)}^{2}}$$

The scaling behavior of *K(τ)* computed from Ulysses data collected outside the ecliptic, close to 2.5 AU, shows three different regimes, which will be further used in the rank-ordered multifractal analysis.

**Figure 4:**
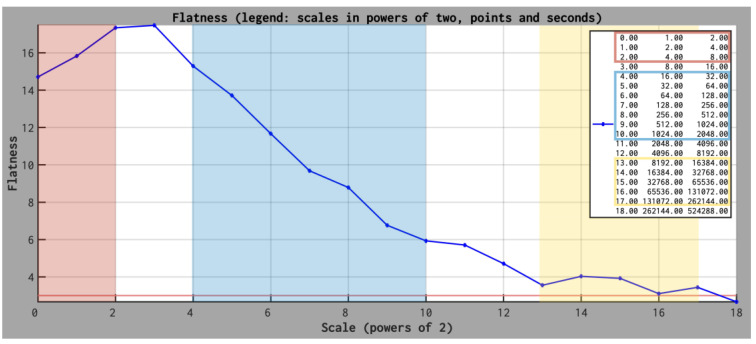
The flatness parameter is computed for the entire time interval and 18 scales, between τ_1_ = 2 s and τ_18_ = 6 days; the scale is specified as “powers of 2” (in order to get the time scales one needs to raise 2 to each value and multiply with the time resolution, δt = 2 s). The three colored ranges emphasize the scales manifesting specific scaling: Range I (marked with red), between τ_1_ = 2 and τ_2_ = 8 s, corresponding to spatial scales roughly equal to 1400 to 5600 km (assuming the Taylor hypothesis is satisfied; the average solar wind speed is 700 km/s) where K(τ) decreases as τ decreases; Range II (marked with blue), between τ_3_ = 32 s and τ_4_ = 2048 s, corresponding to spatial scales roughly equal to 22,400 to 5,734,400 km where K(τ) increases as τ decreases, Range III (marked with yellow) τ_5_ = 4.5 h and τ_6_ = 72.8 h, corresponding to roughly 11.46 to 183.5 millions kilometers. The inset in the top-right indicates the scales considered to compute the flatness, specified in powers of 2 (left column), number of points (central column), and seconds (right column), respectively.

Range I is defined at the smallest scales, between τ_1_ = 2 s and τ_2_ = 8 s, corresponding to spatial scales roughly equal to 1400 to 5600 km, assuming the Taylor hypothesis is satisfied for an average solar wind speed equal to 700 km/s. In this range K(τ) decreases with decreasing scale τ from a maximum value K_max_ recorded for t = 16 s, suggesting the intermittency is “fading” (see Figure 4). Note that this range of scales would correspond to what is called the dissipation range in the standard (spectral/Fourier) description of turbulence. Recent studies on flatness validate this PDF moment as a true statistical descriptor of intermittency [37].

Range II is defined between τ_3_ = 32 s and τ_4_ = 2048 s, corresponding to spatial scales roughly equal to 22,400 to 5,734,400 km (assuming the Taylor hypothesis is satisfied for an average solar wind speed equal to 700 km/s), where the flatness shows a robust increase with decreasing scales, up to its maximum value K_max_ (see Figure 4). This range of scales covers the inertial regime. This is the range where the energy is nonlinearly transferred from larger to smaller scales, as postulated by the classical paradigm of the Richardson cascade of energy. Note that at 1 A.U. the solar wind autocorrelation length is of the order of 1.5 million kilometers [38] but increases in size due to the radial expansion of the solar wind. The flatness behavior suggests the transfer of energy observed by Ulysses is intermittent/fragmented in the inertial range as evidenced by previous studies [39].

Range III is defined between τ_5_ = 4.5 h and τ_6_ = 72.8 h, corresponding to spatial scales roughly equal to 20 to 209 million kilometers (assuming the Taylor hypothesis is satisfied for an average SW speed equal to 700 km/s). In this range the flatness shows a plateau-like trend, taking values close to 3, meaning the fluctuations are random and their distribution is Gaussian (see Figure 4). This is the range of scales where the energy is brought into the system, the fluctuations being completely decorrelated.

**Figure 5:**
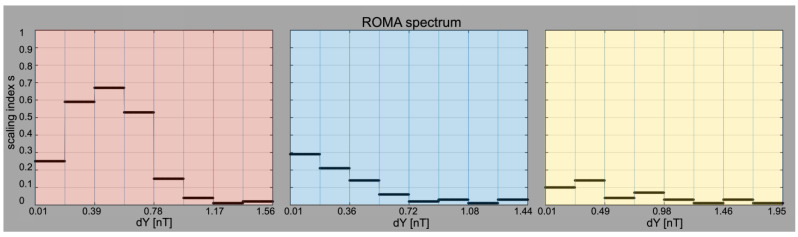
(**left panel**) The full ROMA spectrum computed for magnetic field fluctuations measured by Ulysses in Range I of smallest kinetic scales emphasized in red in Figure 4. (**middle panel**) the ROMA spectrum of magnetic field fluctuations in Range II, inertial of scales emphasized in blue in Figure 4; (**right panel**) the ROMA spectrum of magnetic field fluctuations for Range III, injection of scales emphasized in yellow in Figure 4.

The scaling behavior of *K(τ)* suggests the occurrence of two cross-over scales, one at τ_c1_ = 16 s (approximately 64,000 km) where *K(τ)* takes the maximum value, and the second one at τ_c2_ = 4.5 h (approximately 11.4 million kilometers) which represent the non-Gaussianity threshold, above which the solar wind magnetic fluctuations become Gaussian. The results of the rank-ordered multifractal analysis applied to each of these ranges are shown in Figure 5.

*ROMA spectrum for Ulysses solar wind data, Range I of scales.* The scaled fluctuations, *Y*, take values between 0 and 1 nT which is split into 8 bins for which we compute the ROMA spectrum using the INA Approach. We notice the ROMA *s* index shows a large variability and takes values between *s =* 0.68 and *s =* 0.05 *(*the left panel of Figure 5). The ROMA spectrum can be understood by conceptual elements similar to the Hurst exponent [40]. The smaller fluctuations Y are characterized by an *s* index, which takes values larger than 0.5, meaning the fluctuations manifest persistency, i.e., a long memory of the *fluctuations value* [41]. This behavior is somewhat expected as *Range I* includes scales typical for the kinetic regime. However, for Y > 0.78, the fluctuations are characterized by an *s* index taking values less than 0.5, which can mean anti-persistency, i.e., a long-range memory of *switching* between high and low values [41].

*ROMA spectrum for Ulysses solar wind data, Range II of scales.* At these intermediate, inertial-like scales, the full ROMA spectrum manifests a monotonically decreasing trend. The s index is everywhere smaller than 0.5, suggesting the process is anti-persistent and takes values that decrease with increasing Y (middle panel Figure 5). Such a trend is considered a signature of fully developed intermittent turbulence resulting from nonlinear interactions between coherent structures [1]. Note also that in this range the ROMA spectrum is rather flat for Y > 0.72.

*ROMA spectrum for Ulysses solar wind data, Range III of scales.* At these large, injection-like scales the full ROMA spectrum is flat with rather small values of s and weak variability with Y (right panel of Figure 5). In this range, the ROMA spectrum suggests self-similar, mono-fractal topology of magnetic fluctuations. Such behavior can be generated by fully uncorrelated fluctuations, typical for scales much larger than the solar wind’s autocorrelation length.

### 3.2. ROMA and Solar Wind Turbulence and Complexity at 0.72 AU, in the Ecliptic Plane

We selected magnetic field measurements from Venus Express collected in similar conditions with Ulysses, i.e., at solar minimum observed at approximately 0.72 AU on 28 January 2007. However, the solar wind observed by VEX is slow, with an average speed of the order of 400 km/s. The data set from Venus Express includes 19,000 samples, which ensures moments accuracy up to order *q = 3*, following Dudok de Wit et al. [18]. Nevertheless, a comparison between the ROMA spectra for orders less and equal to 3 and 5, respectively, shows a close similarity; therefore, we include in the manuscript calculations for the same range of orders *q* for both spacecraft data, Ulysses and Venus Express. As we performed for Ulysses data analysis, we attempted to identify ranges of scales and cross-overs from the flatness scale behavior. However, *K(τ)* computed from Venus Express data shows a different scale behavior than the one found from Ulysses data. Indeed, the flatness computed at 0.72 AU for the squared magnetic field does not show a local maximum for an intermediate scale.

**Figure 6:**
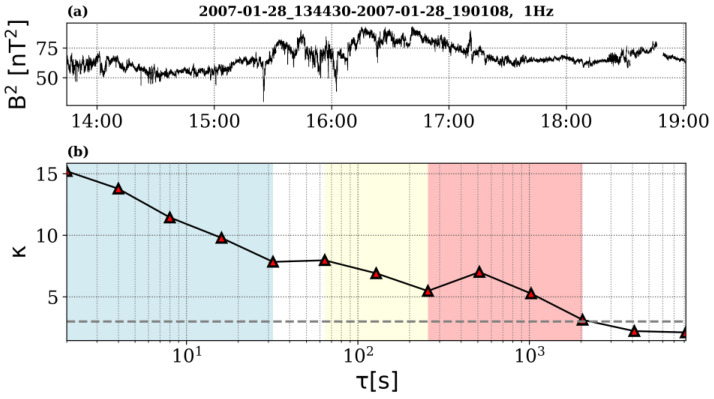
(**a**) Magnetic field energy, ${\left|B\right|}^{2}$, measured by Venus Express in the solar wind on 28 January 2007, between 13:44:30 UT and 19:01:08 UT. (**b**) the flatness computed for B^2^; three ranges of scales are illustrated, between 2 and 32 s (marked with blue), 64 and 256 s (marked with yellow), 256 and 2048 s (marked with red), respectively. The three ranges exhibit different ROMA spectra as discussed in the text.

However, we were able to identify ranges of scales with different characteristics based on flatness analysis, which identified three ranges of scales with the same scale behavior per range and different topological properties derived from ROMA (see Figure 6). The first range of scales is found between τ_1_ = 1 s and τ_2_ = 32 s (corresponding to spatial scales between 400 and 12,800 km, if the Taylor hypothesis is satisfied and an average SW speed is equal to 400 km/s). In this range *K(τ)* follows a power law behavior and increases with decreasing scale, indicating stronger intermittency at the smallest scales. Spectral analysis of the signal [42] indicates the power spectral density becomes steeper for frequencies between 0.2 and 0.6 Hz. A second range is defined between τ_3_ = 64 s and τ_4_ = 256 s (corresponding to spatial scales between 25,600 and 102,400 km), while a third range is identified between τ_3_ = 256 s and τ_4_ = 2048 s (corresponding to spatial scales between 102,400 and 819,200 km). The Fourier analysis for these ranges does not provide meaningful insight into their nature. The main rank-ordered multifractal characteristics of these ranges are discussed below. Note also that, since the time series recorded by VEX in the solar wind is rather short and the solar wind speed is rather low, we do not cover scales pertaining to the injection range at 0.72 AU.

**Figure 7:**
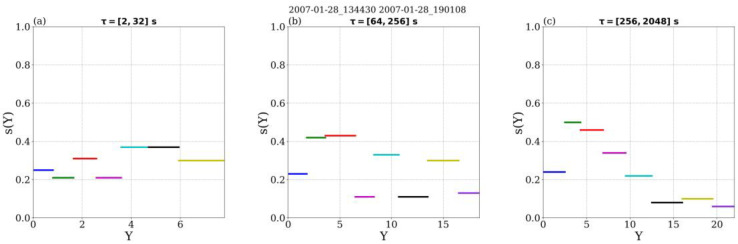
(**a**) Full ROMA spectrum computed for magnetic energy, ${\left|B\right|}^{2}$, measured by Venus Express in the solar wind on 28 January 2007, between 13:44:30 UT and 19:01:08 UT between 1 and 32 s, (**b**) same as (**a**) but for scale range between 64 and 256 s, (**c**) same as (**a**) but for scale range between 256 and 2048 s.

Figure 7 shows the ROMA spectra computed for each of the three ranges discussed above. The ROMA spectrum computed for the smaller range of scales (between τ_1_ = 2 s and τ_2_ = 32 s, see Figure 6, corresponding to spatial scales 400 km and 12,800 km) shows values of the *s* index in a rather limited range, between 0.2 and 0.4, with no clear trend but suggesting anti-persistency. The scaled variable *Y* takes values between 1 and 8 in this scale range, indicating that the level of fluctuations is quite strong, obviously due to higher values of the magnetic field itself at 0.72 AU (compared with data from Ulysses at 2.5 AU). Due to the limited variation of s, we argue the process at the origin of magnetic energy fluctuation in this range of scales is close to self-similarity possibly described by a mono-fractal. The variation of *s* around a mean value of 0.4 can be partially due to limited number of samples of the analyzed ensemble.

The ROMA spectrum computed for the second range of scales defined for VEX data in the solar wind (between τ_3_ = 64 s and τ_4_ = 256 s, see Figure 6, corresponding to spatial scales between 25,600 km and 102,400 km) is shown in panel b of Figure 7 and presents significant differences compared to the one at the smaller scales discussed above. Note also that the range of *Y*, the scaled variable, is larger, between 1 and 20 nT. The *s* index shows a bimodal-like distribution: it takes values around 0.35 and around 0.1, respectively. The two values are intertwined within the full ROMA spectrum, suggesting 2 competing processes are at work simultaneously in this range of scales, each of them possibly self-similar and characterized by a Hurst exponent equal to the observed dominant mean values of *s* (0.35 and 0.1). Note also that the expected value for one of the distributions (*s* = 0.35) is close to the mean value of *s* obtained for the smaller range of scales. Moreover, the second value (*s* = 0.1) is close to the dominant ROMA *s* value obtained for larger scales (see below). A possible interpretation of this result is that in this range of scales, we observe a transition regime, where a dominant process at the larger scales competes with a process becoming dominant at the smaller scales.

The ROMA spectrum computed for the third range of scales defined for VEX data in the solar wind, between t_4_ = 256 s and t_5_ = 2048 s, shown in panel (c) of Figure 7, (corresponding to spatial scales between 102,400 km and 819,200 km), has characteristics, that are quite different from the two ROMA spectra for the intermediate range discussed above. The amplitudes of fluctuations are however comparable to the ones observed for the intermediate range of scales. Nevertheless, with the exception of the first bin, the ROMA spectrum s(Y) shows a clear descending trend, from higher values (starting with s = 0.6) for smaller amplitudes to smaller values for larger amplitudes. The s values for the largest fluctuations oscillate slightly around a mean value of s = 0.1. This type of ROMA spectrum is generally assigned to fully developed intermittent turbulence (see, e.g., [43]).

## 4. Summary and Discussion

In this paper, we discuss a multifractal method based on the rank ordering of fluctuations built on an incremental measure of fluctuations applied for the entire range of targeted scales [7]. ROMA allows us to investigate the properties of magnetic turbulence and complexity in the solar wind from two vantage points, one outside the ecliptic at 2.5 AU (from Ulysses data), the other in the ecliptic, close to Venus at 0.72 AU (from Venus Express data). One key advantage of ROMA is that it treats all fluctuations at all considered scales and all moment orders. The spectrum is implicit and reveals the fractal dimension of rank-ordered fluctuations bearing the same topological mono-fractal behavior described by the respective ROMA *s* index. The latter can be interpreted with a conceptual analogy to the Hurst exponent [40,41]. We extract a description of the scale behavior of magnetic fluctuations for two types of solar wind, slow and fast, at 2 different distances from the Sun, at 0.72 AU in the ecliptic and 2.7 AU outside the ecliptic, at the solar minimum. We are able to identify cross-over scales, based also on a correlative analysis with the flatness behavior, marking a significant change of the multifractal spectrum, as indicated in Figure 4, Figure 5, Figure 6 and Figure 7.

The procedure assumed to calculate the ROMA spectrum is equivalent to a full rescaling of the probability density functions for all scales by considering a local one-parameter rescaling-like rule as in Equation (4), with *s* being the ROMA solution depending on the scaled sizes of fluctuations, $s=s\left(Y\right)$ with $Y=\frac{\delta B}{\tau^{s}}$. To the best of our knowledge, there is only one previously published ROMA analysis of solar wind turbulence [44]. This previous analysis presents one ROMA spectrum computed from wind data at 1 AU for one single range of scales in the inertial range and shows a monotonic trend with s decreasing with increasing values of Y in the anti-persistent range. In Teodorescu et al. [32], we analyzed magnetic field data measured by cluster in the Earth’s magnetosheath for two ranges of scales, smaller (in the kinetic regime) and larger (in the inertial regime). We found differences between the ROMA spectra computed for the two ranges. Thus, at larger scales, the ROMA spectrum is flatter and takes values smaller than 0.5, similar to the ROMA spectrum discussed in this analysis for Ulysses data (Regime III).

Indeed, at larger distances from the Sun, Ulysses data reveal a multi-scale structure of turbulence and complexity with two cross-over scales, separating three different scale regimes of turbulence and complexity. At the smaller scales (we called it *Ulysses Regime I*), in the kinetic range, between 1400 and 5600 km (assuming the Taylor hypothesis is satisfied), intermittency is “fading” as *K(τ)* takes smaller values for decreasing scales. In this range, the ROMA spectrum shows a clear signature of persistent behavior. At the largest scales (we called it *Ulysses Regime III*), between 20 and 209 million kilometers, the magnetic fluctuations are uncorrelated and distributed according to a normal distribution (the flatness is roughly equal to three). The ROMA spectrum is flat for this scaling range and suggests the process leading to the fluctuations is self-similar and the fluctuations are anti-persistent with long memory for switching between higher and lower values. At the intermediate range of scales (we called it *Ulysses Regime II*), between 22,400 and 5,734,400 km, data analysis suggests strong intermittency, with *K(τ)* taking larger and larger values for smaller and smaller scales. The ROMA spectrum shows a decreasing trend with the *s(Y)* index taking smaller values for larger values of Y. Such behavior is generally assigned to developed intermittent turbulence.

At smaller distances from the Sun, in the vicinity of Venus, solar wind data from Venus Express reveal a different picture of turbulence and complexity compared to the one derived from Ulysses. We identified three ranges of scales based on the ROMA analysis. For the first scale range, between 400 and 12,800 km, ROMA indicates the fluctuations are mainly anti-persistent and the s index shows fluctuations around s = 0.35. In this range, we found strong intermittency with *K(τ)* increasing for smaller and smaller scales; the maximum value of *K(τ)* is obtained for the smallest scale. At the largest scales, between 102,400 km and 819,200 km, the ROMA spectrum indicates the presence of fully developed turbulence. Intermittency is also present, confirmed by values of *K(τ)* larger than three and increasing (slightly) with decreasing scales. At the intermediate range of scales, between 25,600 km and 102,400 km, we obtain a bimodal ROMA spectrum suggesting the turbulence, dominant at larger scales, competes with kinetic processes, dominant at the smaller scales.

The picture revealed by our analysis confirms the structure of solar wind magnetic field fluctuations is quite complex. The scale behavior and topology change with distance from the Sun. On the one hand, the radial expansion of the solar wind can have an impact as it implies a dilation of scales with increasing radial distances. On the other hand, the physical processes at work, particularly at the smallest scales where dissipation takes place, can be characterized by different singularities resulting from the dominance of nonlinear interactions changing with the distance from the Sun.

## Data Availability

The data used in this study are available from the FP7 STORM database, https://storm-fp7.eu/index.php/targeted-databases, accessed on 15 March 2024.
